# Supplementary material for: Effect of Special Low-Protein Foods Consumption in the Dietary Pattern and Biochemical Profile of Patients with Inborn Errors of Protein Metabolism: Application of a Database of Special Low-Protein Foods
Source: Nutrients. 2023 Aug 6;15(15):3475. doi: 10.3390/nu15153475 (PMC10421475; doi:10.3390/nu15153475)
Supplement: Supplementary file 1 [file nutrients-15-03475-s001.zip › nutrients-2503071-supplementary.pdf]

Table S1. Characteristics of participants according to specific disorder of protein and amino acid metabolism. [a]

	PKU (n=30)	PKU-BH4 (n=9)	HCU (n=4)	HTI (n=3)	MSUD (n=2)	OAA (n=4)	UCD (n=4)	Transplanted (2 UCD – 1OAA) (n=3)
Age, years	10.4 (3.6 – 17.02)	3.9 (2.1 – 14.7)	7.7 (2.0 – 11.1)	0.9 (0.9 – 7.6)	9.2 (7.9 – 10.5)	9.2 (2.0 – 11.5)	12.9 (5.3 – 15.5)	6.6 (5.4 – 8.9)
Females, n (%)	17 (57)	4 (44)	2 (50)	0 (0)	1 (50)	1 (25)	1 (25)	1 (33)
**Anthropometric Parameters (Z-score)**								
Z-score weight	0.1 (-1.9 – 4.7)	0.3 (-1.5 – 1.5)	–0.8 (-2.9 – 1.6)	–0.3 (-0.4 – –0.2)	0.4 (0.4 – 0.5)	–0.8 (-1.8 – 1.0)	–0.6 (-0.7 – 0.7)	–1.7 (-2.4 – 0.7)
Z-score height	–0.7 (-2.9 – 1.7)	0.2 (-2.4 – 1.4)	0.8 (-3.5 – 2.8)	–2.0 (-2.4 – –1.1)	–1.2 (-1.8 – –0.6)	–1.0 (-2.9 – 1.3)	–0.5 (-2.1 – 0.6)	–2.9 (-3.7 – 0.0)
Z-score BMI	1.06 (-2.0 – 4.8)	0.20 (-2.2 – 3.3)	-0.13 (-3.4 – 0.6)	1.03 (0.7 – 1.9)	1.62 (1.1 – 2.1)	-0.27 (-1.8 – 0.6)	-0.10 (-0.7 – 0.5)	-0.01 (-1.4 – 1.1)
**Plasma biochemical analysis (mg/dL)**								
Glucose, mg/dl	86 (70 – 103)	83 (72 – 128)	85 (70 – 101)	81 (80 – 95)	83 (80 – 85)	96 (65 – 104)	89 (70 – 94)	86 (86 – 87)
TAG, mg/dl	65 (36 – 150)	56 (40 – 130)	68 (34 – 90)	80 (70 – 100)	79 (78 – 79)	82 (67 – 129)	53 (29 – 58)	102 (73 – 102)
TC, mg/dl	134 (95 – 186)	136 (120 – 190)	137 (120 – 166)	120 (104 – 120)	157 (147 – 166)	203 (148 – 222)	142 (100 – 209)	171 (121–248)
HDL-C mg/dl	51 (33 – 84)	50 (33 – 60)	56 (46 – 70)	52 (39 – 55)	57 (53 – 61)	65 (53 – 72)	51 (45 – 85)	50 (45 – 68)
LDL-C mg/dl	68 (49 – 120)	76 (52 – 115)	76 (63 – 83)	70 (53 – 70)	84 (70 – 98)	126 (62 – 152)	82 (55 – 113)	121 (76 – 165)

[a] Values are median (min - max) or *n* (%). BMI, body mass index; HCU, classical homocystinuria; HDL-C, high-density lipoprotein cholesterol; HTI, hereditary tyrosinaemia type I; LDL-C, low-density lipoprotein cholesterol; MSUD, maple syrup urine disease; OAA, organic acidaemias; PKU, phenylketonuria; PKU – BH4, phenylketonuria with tetrahydrobiopterin treatment; TAG, triglycerides; TC, total cholesterol; UCD, urea cycle disorders.

Table S2. Energy and macronutrient intake from the dietary data by group of disorder. [a]

	PKU (n=30)	PKU-BH4 (n=9)	HCU (n=4)	HTI (n=3)	MSUD (n=2)	OAA (n=4)	UCD (n=4)	Transplanted (2 UCD – 1OAA) (n=3)
Energy, Kcal/day	2235 (1398 – 3344)	1581 (1131 – 2768)	1876 (1179 – 2614)	1652 (1591 – 2596)	2462 (1904 – 3019)	2411 (1297 – 2712)	2062 (1563 – 2589)	1468 (1431 – 1761)
Protein, g/day	73.8 (31.6 – 128.9)	36.0 (16.8 – 99.5)	54.4 (24.7 – 70.4)	30.5 (27.5 – 60.3)	57.9 (46.4 – 69.4)	45.9 (19.2 – 63.2)	44.4 (26.0 – 52.5)	33.4 (28.8 – 36.0)
Protein, %	13.2 (8.2 – 20.7)	8.2 (5.8 – 19.7)	10.8 (8.4 – 12.8)	7.7 (6.7 – 9.3)	9.5 (9.2 – 9.7)	7.4 (5.9 – 9.8)	8.2 (7.1 – 9.0)	8.0 (7.6 – 9.8 0)
Natural protein, g/day [U]	12.4 (5.7 – 22.2)	18.8 (9.4 – 25.2)	16.8 (8.1 – 25.6)	7.9 (7.6 – 12.4)	17.6 (16.2 – 19.0)	24.4 (9.1 – 29.6)	38.7 (26.0 – 44.9)	33.4 (28.8 – 36.0)
Natural Protein, %	2.2 (0.9 – 4.4)	5.1 (2.2 – 6.8)	3.4 (2.7 – 4.7)	1.9 (1.9 – 1.9)	3.0 (2.5 – 3.4)	3.8 (2.8 – 5.1)	7.7 (5.2 – 9.0)	8.0(7.6 – 9.8)
Carbohydrates, g/day	309.9 (152.5 – 496.3)	218.9 (127.7 – 367.4)	226.6 (155.9 – 364.9)	217.6 (206.9 – 384.0)	337.4 (303.0 – 371.9)	339.7 (178.3 – 361.4)	266.5 (183.4 – 370.2)	188.8 (175.9 – 232.2)
Carbohydrates, %	53.8 (40.6 – 63.0)	52.9 (44.0 – 62.7)	53.0 (41.5 – 55.8)	54.7 (50.1 – 59.2)	56.5 (49.3 – 63.6)	54.4 (53.3 – 59.4)	53.7 (45.2 – 57.6)	51.5 (49.2 – 52.8)
Sugars, g	72.6 (14.8 – 142.1)	56.0 (25.0 – 91.8)	74.9 (27.9 – 102.8)	64.2 (59.1 – 65.8)	78.7 (76.0 – 81.4)	79.7 (50.7 – 108.8)	57.8 (33.2 – 70.2)	53.4 (36.5 – 79.0)
Sugars, %	12.5 (3.4 – 24.9)	13.9 (8.5 – 24.1)	16.4 (7.4 – 18.2)	14.9 (10.1 – 15.6)	13.6 (10.1 – 17.1)	14.6 (12.8 – 16.1)	11.0 (7.1 – 14.2)	14.6 (10.2 – 17.9)
Fiber, g/day	19.0 (8.9 – 58.5)	18.8 (10.8 – 61.5 3)	20.1 (10.1 – 24.3)	23.0 (17.0 – 23.9)	42.5 (39.7 – 45.4)	19.5(6.5 – 35.3)	12.6 (8.8 – 35.1)	19.0 (4.8 – 19.4)
Starch, g/day	5.2 (0 – 75.4)	14.5 (2.0 – 25.6)	5.6 (1.9 – 23.4)	4.3(2.9 – 8.1)	25.0 (4.3 – 45.8)	21.8 (8.5 – 45.3)	19.4 (2.0 – 38.6)	23.2 (12.9 – 30.2)
Total fat, g/day	76.3 (52.7 – 111.7)	59.6 (35.0 – 99.8)	83.5 (48.2 – 96.7)	79.4 (65.5 – 91.0)	95.1 (52.3 – 137.9)	93.8 (55.4 – 114.0)	88.6 (68.3 – 98.4)	67.9 (62.6 – 76.6)
Total fat, %	31.8 (22.8 – 42.3)	33.9 (25.6 – 48.0)	36.5 (33.3 – 45.8)	37.1 (31.6 – 43.2)	32.9 (24.7 – 41.1)	37.2 (33.3 – 38.4)	38.1 (33.1 – 45.1)	39.1 (38.4 – 42.7)
SFA, %	8.2 (4.1 – 13.3)	8.6 (5.1 – 13.8)	11.1 (8.6 – 16.1)	10.3 (8.2 – 14.7)	8.9 (6.7 – 11.2)	11.3 (7.7 – 12.9)	9.8 (6.1 – 15.4)	11.7 (9.4 – 13.3)
MUFA, %	13.4 (7.5 – 22.1)	17.2 (11.0 – 24.1)	14.8 (13.3 – 18.8)	17.8(15.1 – 19.0)	17.3(12.6 – 22.0)	16.9 (14.8 – 19.2)	16.2(14.3 – 18.7)	19.5 (18.2 – 21.0)
PUFA, %	4.0 (1.7 – 6.1)	4.0 (2.8 – 5.0)	3.3 (2.2 – 4.7)	6.7 (6.2 – 6.9)	4.1 (2.8 – 5.5)	4.0 (2.8 – 4.6)	6.4 (4.0 – 9.6)	4.0 (3.8 – 4.6)
Cholesterol, g/day	10.6 (0 – 151.5)	71.5 (21.7 – 172.3)	51.5 (19.0 – 132.3)	10.8 (0 – 79.1)	31.9 (31.0 – 32.8)	131.1 (14.3 – 191.5)	117.8 (85.4 – 143.8)	88.8 (63.4 – 101.8)

[a] All values are in median (min - max). [U] The natural protein included high biological value protein and also that from fruits, vegetables and potatoes.

HCU, classical homocystinuria; HTI, hereditary tyrosinaemia type I; MSUD, maple syrup urine disease; MUFA, monounsaturated fatty acids; OAA, organic acidaemias; PFAAs, precursor-free L-amino acid supplement; PKU, phenylketonuria; PKU – BH4, phenylketonuria with tetrahydrobiopterin treatment; PUFA, polyunsaturated fatty acids; SFA, saturated fatty acids; UCD, urea cycle disorders.

Table S3. Special low-protein foods (SLPFs) intake by group of disorder. [a]

	PKU (n=30)	PKU-BH4 (n=9)	HCU (n=4)	HTI (n=3)	MSUD (n=2)	OAA (n=4)	UCD (n=4)	Transplanted (2 UCD – 1OAA) (n=3)
Energy Kcal/day	613 (188 – 1363)	378 (50 – 849)	585 (166 – 1140)	413 (190 – 703)	725 (675 – 774)	643 (377 – 1209)	347 (164 – 599)	579 (215 – 638)
Energy, %	30 (13 – 53)	25 (4 – 36)	30 (11 – 44)	25 (12 – 27)	31 (26 – 36)	34 (17 – 53)	18 (6 – 31)	33 (15 – 45)
Protein, g/day	2.1 (0.4 – 6.0)	1.4 (0.3 – 6.3)	1.6 (0.3 – 6.5)	1.5 (0.2 – 2.6)	2.8 (2.2 – 3.3)	1.7 (0.9 – 3.8)	1.1 (0.1 – 3.0)	0.0 (0.0 – 3.2)
Protein, %	1.4 (0.3 – 5.6)	1.7 (0.9 – 7.1)	0.9 (0.6 – 2.1)	1.5 (0.2 – 3.2)	1.5 (1.4 – 1.7)	1.1 (0.6 – 1.4)	0.9 (0.0 – 2.1)	0.0 (0.0 – 2.1)
Carbohydrates, g/day	121.8 (30.0 – 251.8)	58.0 (7.7 – 167.9)	108.8 (36.9 – 209.8)	64.4 (43.4 – 111.4)	131.0 (129.3 – 132.8)	103.8 (84.4 – 179.7)	70.2 (39.2 – 97.9)	81.7 (26.2 – 96.4)
Carbohydrates, %	24.2 (8.4 – 37.9)	19.2 (2.7 – 25.2)	22.6 (9.8 – 32.1)	15.6 (10.9 – 17.2)	22.4 (17.6 – 27.2)	20.8 (14.7 – 34.7)	16.1 (6.1 – 20.0)	21.9 (7.1 – 22.8)
Sugars, g	13.2 (0.2 – 64.7)	8.5 (0.7 – 23.4)	18.5 (0.2 – 38.8)	9.3 (7.4 – 14.9)	21.2 (19.6 – 22.8)	24.3 (0.7 – 37.8)	5.6 (0.4 – 14.7)	22.1 (11.3 – 27.9)
Sugars, %	2.6 (0.03 – 10.7)	1.5 (0.2 – 7.5)	3.3 (0.1 – 6.2)	1.9 (1.4 – 3.6)	3.7 (2.6 – 4.8)	4.5 (0.1 – 8.0)	1.2 (0.1 – 3.0)	5.0 (3.1 – 7.8)
Fiber, g/day	4.8 (0.0 – 26.0)	1.1 (0.7 – 5.7)	6.6 (0.1 – 14.2)	1.5 (0.1 – 2.3)	4.7 (2.5 – 6.9)	0.7 (0.0 – 1.1)	1.5 (0.0 – 25.6)	0.0 (0.0 – 3.2)
Starch, g/day	0.0 (0.0 – 43.7)	0.0 (0.0 – 21.9)	0.0 (0.0 – 0.0)	0.0 (0.0 – 0.0)	0.00 (0.0 – 0.0)	0.0 (0.0 – 6.5)	0.0 (0.0 – 0.0)	0.0 (0.0 – 14.6)
Total fat, g/day	17.1 (0.7 – 38.5)	8.3 (1.1 – 20.3)	15.9 (1.9 – 43.7)	17.6 (1.2 – 27.5)	21.1 (16.6 – 25.6)	24.6 (4.0 – 52.8)	17.8 (0.6 – 22.5)	20.1 (12.3 – 31.4)
Total fat, %	7.3 (0.3 – 14.2)	5.5 (0.7 – 14.9)	6.9 (1.2 – 15.1)	9.5 (0.7 – 9.6)	7.7 (7.6 – 7.8)	12.9 (1.6 – 17.5)	8.8 (0.2 – 10.2)	10.3 (7.5 – 19.8)
SFA, %	3.2 (0.1 – 9.2)	3.5 (0.1 – 8.5)	3.6 (0.6 – 5.5)	4.2 (0.2 – 4.7)	4.0 (3.9 – 4.1)	5.5 (0.7 – 9.3)	3.1 (0.1 – 5.4)	3.8 (3.5 – 7.0)
MUFA, %	0.7 (0.0 – 3.9)	0.0 (0.0 – 3.5)	1.1 (0.01 – 2.4)	2.8 (0.04 – 3.7)	2.0 (1.8 – 2.3)	3.3 (0.6 – 5.6)	1.4 (0.0 – 3.2)	2.6 (1.7 – 8.6)
PUFA, %	0.2 (0.0 – 1.7)	0.0 (0.0 – 1.0)	0.2 (0.01 – 0.4)	0.4 (0.1 – 1.2)	0.5 (0.5 – 0.6)	0.8 (0.1 – 1.6)	0.8 (0.01 – 2.1)	0.4 (0.3 – 2.1)
Cholesterol, g/day	0.0 (0.0 – 10.2)	0.0 (0.0 – 5.0)	0.3 (0.0 – 4.3)	0.0 (0.0 – 0.0)	12.7 (0.0 – 25.5)	0.0 (0.0 – 7.7)	0.0 (0.0 – 21.7)	0.0 (0.0 – 0.0)

[a] All values are in median (min - max).

HCU, classical homocystinuria; HTI, hereditary tyrosinaemia type I; MSUD, maple syrup urine disease; MUFA, monounsaturated fatty acids; OAA, organic acidaemias; PFAAs, precursor-free L-amino acid supplement; PKU, phenylketonuria; PKU – BH4, phenylketonuria with tetrahydrobiopterin treatment; PUFA, polyunsaturated fatty acids; SFA, saturated fatty acids; UCD, urea cycle disorders.

Table S4. Precursor-free L-amino acid supplements (PFAAs) intake by group of disorder. [a]

	PKU (n=30)	PKU-BH4 (n=9)	HCU (n=4)	HTI (n=3)	MSUD (n=2)	OAA (n=4)	UCD (n=4)	Transplanted (2 UCD – 1OAA) (n=3)
Energy Kcal/day	620 (334 – 1135)	237 (0.0 – 1015)	295 (278 – 381)	801 (686 – 880)	706 (381 – 1031)	295 (83 – 844)	0 (0 – 948)	0 (0 – 0)
Energy, %	29 (14 – 52)	16 (0.0 – 37)	19 (11 – 26)	42 (34 – 50)	27 (20 – 34)	13 (6 – 33)	0 (0 – 37)	0 (0 – 0)
Protein, g/day	59.1 (14.6 – 115.6)	19.5 (0.0 – 80.6)	37.5 (16.6 – 44.9)	22.9 (19.7 – 47.9)	40.3 (30.2 – 50.4)	16.8 (10.1 – 42.8)	0.0 (0.0 – 19.0)	0.0 (0.0 – 0.0)
Protein, %	10.3(3.8 – 18.5)	5.1(0.0 – 14.1)	7.4(5.6 – 8.0)	5.8(4.8 – 7.4)	6.5(6.3 – 6.7)	3.2(2.1 – 6.6)	0.0(0.0 – 2.9)	0.0(0.0 – 0.0)
Carbohydrates, g/day	62.3 (12.8 – 129.6)	30.6 (0.0 – 92.2)	27.8 (21.1 – 35.1)	86.6 (74.3 – 130.5)	68.6 (34.6 – 102.6)	33.5 (10.3 – 81.9)	0.0 (0.0 – 164.7)	0.0 (0.0 – 0.0)
Carbohydrates, %	12.2 (2.6 – 59.2)	8.4 (0.0 – 14.0)	6.5 (3.2 – 11.9)	20.1 (18.0 – 21.8)	10.4 (7.3 – 13.6)	5.6 (3.2 – 12.7)	0.0 (0.0 – 25.5)	0.0 (0.0 – 0.0)
Sugars, g	8.8 (0.0 – 32.2)	3.9 (0.0 – 26.3)	13.9 (3.6 – 17.6)	11.1 (0.0 – 13.0)	4.6 (3.5 – 5.8)	6.6 (4.7 – 14.0)	0.0 (0.0 – 0.0)	0.0 (0.0 – 0.0)
Sugars, %	1.6 (0.0 – 6.2)	1.1 (0.0 – 9.2)	2.9 (1.0 – 3.5)	2.7 (0.0 – 3.3)	0.7 (0.7 – 0.8)	1.6 (0.7 – 2.1)	0.0 (0.0 – 0.0)	0.0 (0.0 – 0.0)
Fiber, g/day	0.6 (0.0 – 26.3)	0.3 (0.00 – 32.3)	6.6 (0.0 – 13.1)	8.0 (1.6 – 9.3)	16.1 (12.1 – 20.2)	2.4 (0.0 – 1.1)	0.0 (0.0 – 0.0)	0.0 (0.0 – 0.0)
Starch, g/day	0.0 (0.0 – 0.0)	0.0 (0.0 – 0.0)	0.0 (0.0 – 0.0)	0.0 (0.0 – 0.0)	0.0 (0.0 – 0.0)	0.0 (0.0 – 0.0)	0.0 (0.0 – 0.0)	0.0 (0.0 – 0.0)
Total fat, g/day	15.2 (1.3 – 38.3)	5.9 (0.0 – 36.0)	6.6 (1.6 – 13.5)	34.5 (18.5 – 40.3)	30.0 (13.5 – 46.5)	10.5 (0.1 – 38.3)	0.0 (0.0 – 23.7)	0.0 (0.0 – 0.0)
Total fat, %	6.1 (0.5 – 19.8)	4.5 (0 – 11.7)	4.4 (0.5 – 8.9)	18.8 (6.4 – 22.8)	10.1 (6.4 – 13.9)	4.2 (0.1 – 13.3)	0.0 (0.0 – 8.2)	0.0 (0.0 – 0.0)
SFA, %	1.1 (0.0 – 5.8)	0.7 (0.0 – 1.9)	0.6 (0.0 – 2.9)	6.1 (1.0 – 7.4)	2.4 (1.0 – 3.9)	1.4 (0.0 – 3.7)	0.0 (0.0 – 1.2)	0.0 (0.0 – 0.0)
MUFA, %	2.2 (0.0 – 11.0)	1.7 (0.0 – 7.7)	1.8 (0.0 – 5.3)	7.7 (1.4 – 9.3)	5.5 (4.2 – 6.9)	1.7 (0.0 – 6.7)	0.0 (0.0 – 1.8)	0.0 (0.0 – 0.0)
PUFA, %	1.9 (0.0 – 11.1)	0.9 (0.0 – 2.6)	0.6 (0.0 – 1.9)	4.1 (3.7 – 5.0)	1.6 (0.9 – 2.3)	0.9 (0.0 – 2.2)	0.0 (0.0 – 4.8)	0.0 (0.0 – 0.0)
Cholesterol, g/day	0.0 (0.0 – 0.0)	0.0 (0.0 – 0.0)	0.0 (0.0 – 0.0)	0.0 (0.0 – 0.0)	0.0 (0.0 – 0.0)	0.0 (0.0 – 0.0)	0.0 (0.0 – 0.0)	0.0 (0.0 – 0.0)

[a] All values are in median (min - max). HCU, classical homocystinuria; HTI, hereditary tyrosinaemia type I; MSUD, maple syrup urine disease; MUFA, monounsaturated fatty acids; OAA, organic acidaemias; PKU, phenylketonuria; PKU – BH4, phenylketonuria with tetrahydrobiopterin treatment; PUFA, polyunsaturated fatty acids; SFA, saturated fatty acids; UCD, urea cycle disorders.

Table S5. Characteristics of study participants, dietary intake, and plasma biochemical parameters by tertiles of percentage of SLPFs consumption. [1]

	**Tertiles of SLPFs consumption**			
	**1**<br>**< 24 %**<br>**(n = 19)**	**2**<br>**24 – 32 %**<br>**(n = 20)**	**3**<br>**> 32 %**<br>**(n = 20)**	***P***
Age, *years*	7.4 (3.7 – 11.1)	8.9 (5.2 – 12.6)	10.7 (7.7 – 14.2)	0.17
Females, *n* (%)	6 (32)	11 (55)	10 (50)	0.31
**Anthropometric parameters**				
Weight Z-score	-0.3 (–0.9 – 0.6)	0.1 (–0.8 – 0.6)	0.3 (–0.6 – 0.8)	0.52
Height Z-score	-1.0 (–2.1 – 0.3)	-0.8 (–1.2 – 0.6)	-0.5 (–2.0 – 0.2)	0.76
BMI Z-score	0.5 (–0.03 – 0.9)	0.7 (–0.6 – 1.1)	1.1 (–0.5 – 1.4)	0.59
**Dietary intake**				
Total energy, kcal/day	1591 (1463 – 2161) [b]	2094 (1770 – 2556) [a]	2362 (1758 – 2590) [a]	0.013
Energy from SLPFs, %	14.8 (12.0 – 19.7) [c]	28.1 (25.7 – 30.6) [b]	39.7 (35.1 – 43.7) [a]	<0.001
Energy from PFAAs, %	28.7 (16.4 – 36.4) [ab]	32.6 (23.0 – 36.2) [a]	20.5 (7.6 – 28.3) [b]	0.043
Total protein, %	9.8 (8.1 – 13.7)	9.9 (9.0 – 14.1)	11.5 (8.5 – 15.1)	0.56
Natural Protein, %	4.4 (3.0 – 5.3) [a]	2.1 (1.9 – 3.7) [b]	2.4 (1.9 – 3.8) [b]	0.015
Carbohydrates, %	53.1 (50.1 – 57.2)	53.0 (50.7 – 58.7)	54.0 (52.8 – 57.1)	0.59
Sugars, %	13.2 (11.7 – 14.9)	11.6 (8.5 – 15.5)	15.8 (12.0 – 17.1)	0.058
Fiber, g/day	19.0 (12.9 – 26.4)	19.4 (16.7 – 32.2)	18.8 (14.9 – 24.9)	0.63
Total fats, %	34.2 (33.1 – 39.1)	33.4 (31.0 – 40.3)	32.7 (29.4 – 37.9)	0.54
SFAs, %	9.2 (6.3 – 10.4)	9.2 (6.6 – 11.4)	8.7 (7.6 – 12.5)	0.78
MUFAs, %	17.1 (14.4 – 18.8)	15.9 (12.3 – 18.4)	13.4 (12.3 – 16.8)	0.11
PUFAs, %	4.6 (3.8 – 5.2) [a]	4.4 (3.4 – 5.8) [a]	3.4 (2.5 – 4.5) [b]	0.016
Cholesterol, mg/day	63.4 (29.4 – 132.9)	31.3 (7.0 – 70.5)	20.3 (6.5 – 54.1)	0.084
**Plasma biochemical parameters**				
Glucose, mg/dL	85.0 (81.0 – 94.0)	84.0 (81.0 – 89.0)	86.0 (78.0 – 92.0)	0.65

Triglycerides, mg/dL	63.0 (53.0 – 92.0)	64.0 (44.5 – 76.8)	73.5 (60.3 – 89.8)	0.20
Total Cholestrol, mg/dL	127.0 (121.0 – 182.0)	133.5 (116.3 – 150.3)	149.5 (124.8 – 167.5)	0.32
HDL-C, mg/dL	51.0 (48.0 – 56.0)	52.0 (41.3 – 60.0)	52.0 (44.8 – 59.5)	0.99
LDL-C mg/dL	76.0 (63.0 – 112.0)	68.5 (58.8 – 86.8)	80.5 (58.3 – 97.3)	0.36

[1] All values are median (P25 - P75) or n (%). Medians in a row with superscripts without a common letter differ, $P < 0.05$. Data analyzed by Kruskal–Wallis and Mann–Whitney post-hoc test or Chi square test.

Table S6. Characteristics of plasma biochemical analysis by tertiles of percentage of dairy SLPF consumption. [1,2]

**Plasma Biochemical Analysis (mg/dL)**	**1**<br>**< 1.0%**<br>**(n = 19)**	**2**<br>**1.0 – 5.2 %**<br>**(n = 20)**	**3**<br>**> 5.2 %**<br>**(n = 20)**	p
Glucose				
Normal glucose levels (< 100 mg/dL), median (IQR), [*n (%)*]	85.0 (81.5 – 91.5)<br>[16 (84)]	83.0 (77.0 – 86.0)<br>[19 (95)]	86.0 (80.3 – 93.5)<br>[20 (100)]	0.39
Prediabetes risk glucose levels (100–125 mg/dL), median (IQR), [*n (%)*]	103.5 (103.0; 104.0) [2]<br>[2 (11)]	101.0<br>[1 (5)]	[0 (0)]	0.22
Type 2 diabetes risk glucose levels (>125 mg/dL), median (IQR), [*n (%)*]	128.0<br>[1 (5)]	[0 (0)]	[0 (0)]	-
Triglycerides				
Acceptable TAGs values (<75 mg/dL) (under 9 y), median (IQR), [*n (%)*]	63.0 (46.0 – 67.0)<br>[5 (50)]	52.0 (38.0 – 66.0)<br>[8 (80)]	60.0 (53.0 – 70.0)<br>[7 (70)]	0.64
Borderline TAGs values (75–99 mg/dL) (under 9 y), median (IQR), [*n (%)*]	79.0 (78.0; 80.0) [2]<br>[2 (20)]	84.5 (79.0; 90.0) [2]<br>[2 (20)]	90.0<br>[1 (10)]	0.40
High TAGs values (>100 mg/dL) (under 9 y), median (IQR), [*n (%)*]	121.0 (100.0; 130.0) [2]<br>[3 (30)]	[0 (0)]	102.0 (102.0; 102.0) [2]<br>[2 (20)]	0.55
Acceptable TAGs values (<90 mg/dl) (10-19 y), median (IQR), [*n (%)*]	58.0 (47.8 – 67.0)<br>[8 (89)]	50.0 (45.0 – 69.0)<br>[7 (70)]	71.5 (51.8 – 86.5)<br>[8 (80)]	0.25
Borderline TAGs values (90–129 mg/dL) (10-19 y), median (IQR), [*n (%)*]	92.0<br>[1 (11)]	109.0<br>[1 (10)]	118.5 (108.0; 129.0) [2]<br>[2 (20)]	0.41
High TAGs values (>130 mg/dL) (10-19 y), median (IQR), [*n (%)*]	[0 (0)]	146.0 (142.0; 150.0) [2]<br>[2 (20)]	[0 (0)]	-
Total Cholesterol				
Acceptable TC values (<170 mg/dL), median (IQR), [*n (%)*]	121.0 (120.0 – 133.0) [b]<br>[15 (79)]	123.5 (115.8 – 145.0) [b]<br>[18 (90)]	148.0 (130.5 – 164.5) [a]<br>[13 (65)]	0.014
Borderline TC values (170–199 mg/dL), median (IQR), [*n (%)*]	190.0 (182.0; 199.0) [2]<br>[3 (16)]	171.0 (170.0; 172.0) [2]<br>[2 (10)]	172.5 (171.0 – 183.0)<br>[4 (20)]	0.09
High TC values (≥200 mg/dL), median (IQR), [*n (%)*]	209.0<br>[1 (5)]	[0 (0)]	222.0 (207.0; 248.0) [2]<br>[3 (15)]	0.66

HDL-C				
Acceptable HDL-C values (>45 mg/dL), median (IQR), [*n (%)*]	55.0 (51.0 – 60.0) [a] [15 (79)]	50.0 (48.0 – 53.0) [b] [15 (75)]	59.0 (51.5 – 68.5) [a] [18 (90)]	0.026
Borderline HDL-C values (40–45 mg/dL), median (IQR), [*n (%)*]	[0 (0)]	43.5 (43.0; 44.0) [2] [2 (10)]	43.0 [1 (5)]	0.48
Low HDL-C values (<40 mg/dl), median (IQR), [*n (%)*]	36.5 (33.8 – 39.3) [4 (21)]	34.0 (33.0; 40.0) [2] [3 (15)]	39.0 [1 (5)]	0.77
LDL-C				
Acceptable LDL-C values (<110 mg/dL), median (IQR), [*n (%)*]	63.0 (57.0 – 70.0) [b] [15 (79)]	69.5 (56.5 – 86.8) [ab] [20 (100)]	82.0 (70.0 – 95.0) [a] [15 (75)]	0.015
Borderline LDL-C values (110–129 mg/dL), median (IQR), [*n (%)*]	114.0 (112.3 – 122.5) [4 (21)]	[0 (0)]	121.0 (120.0; 127.0) [2] [3 (15)]	0.16
High LDL-C values (≥130 mg/dL), median (IQR), [*n (%)*]	[0 (0)]	[0 (0)]	158.5 (152.0; 165.0) [2] [2 (10)]	-

[1] All values are median (IQR), [n (%)]. Medians in a row with superscripts without a common letter differ, $p < 0.05$. Data analysed by Kruskal–Wallis and Mann–Whitney post hoc test. HDL-C, high-density lipoprotein cholesterol; LDL-C, low-density lipoprotein cholesterol; TAGs, triglycerides; TC, total cholesterol.

[2] Values are median (maximum; minimum).

Values for plasma lipid levels are from the NCEP Expert Panel on Cholesterol levels in Children [1, 2]. Values for plasma glucose and TAG levels are from the Screening for Prediabetes and Type 2 Diabetes in Children and Adolescents, US Preventive Services Task Force Recommendation Statement [3].

[1] De Jesus, J.M. Expert Panel on Integrated Guidelines for Cardiovascular Health and Risk Reduction in Children and Adolescents: Summary Report. *Pediatrics* **2011**, *128*, 213–256, doi:10.1542/peds.2009-2107C.

[2] Schefelker, J.M.; Peterson, A.L. Screening and Management of Dyslipidemia in Children and Adolescents. *J. Clin. Med.* **2022**, *11*, doi:10.3390/jcm11216479.

[3] Mangione, C.M.; Barry, M.J.; Nicholson, W.K.; Cabana, M.; Chelmow, D.; Coker, T.R.; Davidson, K.W.; Davis, E.M.; Donahue, K.E.; Jaén, C.R.; et al. Screening for Prediabetes and Type 2 Diabetes in Children and Adolescents: US Preventive Services Task Force Recommendation Statement. *Jama* **2022**, *328*, 963–967, doi:10.1001/jama.2022.14543
